# A Narrative Review on the Advance of Probiotics to Metabiotics

**DOI:** 10.4014/jmb.2311.11023

**Published:** 2024-01-29

**Authors:** Hye Ji Jang, Na-Kyoung Lee, Hyun-Dong Paik

**Affiliations:** Department of Food Science and Biotechnology of Animal Resources, Konkuk University, Seoul 05029, Republic of Korea

**Keywords:** Metabiotics, gut-brain axis, neurological disorder, immunomodulation, anticancer

## Abstract

Recently, the term metabiotics has emerged as a new concept of probiotics. This concept entails combining existing probiotic components with metabolic by-products improve specific physiological functionalities. Representative ingredients of these metabiotics include short-chain fatty acids (SCFAs), bacteriocins, polysaccharides, and peptides. The new concept is highly regarded as it complements the side effects of existing probiotics and is safe and easy to administer. Known health functions of metabiotics are mainly immune regulation, anti-inflammatory, anticancer, and brain-neurological health. Research has been actively conducted on the health benefits related to the composition of intestinal microorganisms. Among them, the focus has been on brain neurological health, which requires extensive research. This study showed that neurological disorders, such as depression, anxiety, autism spectrum disorder, Alzheimer's disease, and Parkinson's disease, can be treated and prevented according to the gut-brain axis theory by changing the intestinal microflora. In addition, various studies are being conducted on the immunomodulatory and anticancer effects of substances related to metabiotics of the microbiome. In particular, its efficacy is expected to be confirmed through human studies on various cancers. Therefore, developing various health functional effects of the next-generation probiotics such as metabiotics to prevent or treatment of various diseases is anticipated.

## Introduction

According to the World Health Organization (WHO) and Food Agriculture Organization (FAO), probiotics are defined as live microorganisms administered in adequate amounts for health benefits on the host [1]. Generally, probiotics have been associated with many health benefits, such as regulating intestinal flora, immune functions, as well as antimicrobial, anticancer, and anti-inflammatory effects [2, 3]. However, various concerns including viability control, gastrointestinal side effects, fungemia, and endocarditis exist [4-6]. Therefore, next-generation probiotics are being investigated to overcome these concerns [5].

Metabiotics are defined as bioactive compounds generated through the metabolic process of probiotic microorganisms, exerting a positive influence on various physiological process [7]. Metabiotics collectively refers to constituents of probiotic microorganisms, their metabolic by–products, and signaling molecules with well-defined chemical compositions [8]. These components are characterized by their ability to enhance specific physiological functions tailored to the host, including regulatory, metabolic, and behavioral responses. These interactions are closely associated with host microbiota activity [9]. The representative components of metabiotics are short-chain fatty acids (SCFAs), polyunsaturated fatty acids (PUFAs), bacteriocins, polysaccharides, and peptides [10].

Thus, this review aims to provide an overview of the metabiotics. We outline the current status of probiotics advancements to metabiotics and their health benefits. We also summarized the functionalities of metabiotics and provide an outlook on their future potential.

## An Advance from Probiotics to Metabiotics

Lactic acid bacteria (LAB), especially those with positive effects on health, are commonly known as probiotics, a term introduced by Nobel Prize winner, Elie Metchnikoff [11]. *Lactobacillus*, *Pediococcus*, *Leuconostoc*, and *Bifidobacterium* are widely used as probiotics [12]. Nevertheless, for application as a probiotic, microorganisms should have nonpathogenic and nonvirulent characteristics in relation to humans [13]. Most probiotic applications have been reported in the prevention and treatment of diverse human diseases or disorders [2, 14]. Typically, probiotics should survive under intestinal conditions of low pH and high concentrations of bile salts, and show a high ability to adhere to intestinal epithelial cells [2, 15]. In addition, probiotics are known to have many health benefits as antioxidants, antivirals, anti-inflammatories, antimicrobials, as well as anticancer, cholesterol lowering, and anticavity drugs [16-19]. Currently, many food and pharmaceutical industries are conducting functional probiotics research to improve health, and probiotic products have become popular globally [20, 21]. In addition to their use in managing various health conditions, probiotics are actively promoted in media reports to consumers as a means of improving or maintaining health [22]. Probiotics are widely available as food products such as dairy products and juices, as well as capsules, and powders [23]. Regarding the final products, the appropriate dosage of probiotics should be based on the efficacy of human clinical trials. Probiotic products should contain at least 10^6^ CFU/ml or g and a daily consumption of approximately 10^8^–10^9^ CFU/ml or g to provide benefits to the consumer [11].

Prebiotics is defined as non-digestible dietary fibers or compounds that serve as food for beneficial microorganisms, particularly probiotics, in the gastrointestinal tract [24, 25]. In 2016, a panel of experts in the field of microbiology, nutrition, and clinical research was convened by the International Scientific Association for Probiotics and Prebiotics (ISAPP) to assess and redefine the scope of prebiotics [26]. Typical substances included in prebiotics are polysaccharides (resistant starch, dextrin, and pectin) and oligosaccharides such as fructooligosaccharides (FOS) and galactooligosaccharide (GOS), xylooligosacharides (XOS), isomaltooligosaccharides (IMO), mannanoligosaccharides (MOS), raffinoseoligosaccharides (RFOs), arabinoxylanoligosaccharides (AXOS), and lactulose [27-29]. Prebiotics has been reported to inhibit pathogenic bacteria and cancer, reduce cardiovascular disease and cholesterol levels, and prevent constipation and obesity [29]. In addition, prebiotics are fermented by microorganisms in the intestines to produce SCFAs, including propionate, butyrate, and acetate, showing various health benefits, such as prevention of gastrointestinal conditions and reduction of cancer [28, 30, 31]. Recently, the significance of gut microbiotas has become increasingly important for health. Consequently, there is an emerging strategy to control microbiotas and their relationship with host [28]. Owing to their characteristics, prebiotics have been used as functional ingredients in various food industries [30]. Significant research on prebiotics has indicated their utilization in functional food applications, which involve providing benefits beyond their inherent nutrient compound to enhance overall well-being [32].

Synbiotics are a combination of probiotics and prebiotics that synergistically improve overall health and well-being [33]. Notably, the beneficial effects of synbiotics are greater than those of probiotics or prebiotics [34]. Recently, the ISAPP has defined synbiotics as follows: “a mixture of viable microorganisms and substrate(s) that are selectively utilized by host microorganisms and confer a health benefit on the host” [35]. Representative functional health effects include the re-modulation of gut bacterial dysbiosis, improvement of immunomodulation, and prevention of obesity. A principal example of synbiotics containing *L. rhamnosus* and *Bifidobacterium lactis* could effectively regulate and reduce pathogenic bacteria under intestinal conditions [36]. Additionally, one study has reported synbiotics containing *Lactobacillus paracasei* (probiotics) and *Opuntia humifusa* (prebiotics) to have improved irritable bowel syndrome (IBS) by lowering tumor necrosis factor alpha (TNF-α), decreasing serum corticosterone levels, and increasing expression of tight junction proteins [37].

Postbiotics are bioactive compounds produced by probiotics during metabolic processes. Unlike probiotics, which are live microorganisms, and prebiotics, which are the substrate that promote the growth of beneficial microorganisms, postbiotics are microbial cell components and actual metabolic products that emerge from probiotic activity, such as secreted enzymes, proteins, vitamins, SCFAs, and phenols [5, 38]. The terms ‘post’, indicating ‘after’, and ‘biotic’, which refers to originate from living organisms’, resulted in ‘postbiotic’. These components together imply ‘after life’, indicating materials that are produced by living microorganisms [39]. In general, postbiotics possess the advantages of stability and convenient transportability, which improve shelf life and simplify packaging. Thus, the active ingredient can be transferred to the desired location in the intestine [40]. Furthermore, postbiotics have a wide range of beneficial effects, including immunomodulatory, anticancer, and antimicrobial effects [41]. As virus-related diseases have increased significantly over the pasts few years, studies of postbiotics, which are effective in immune-modulation, antimicrobial, and antiviral, have been reported [42]. However, the precise of mechanism of postbiotics action is yet to be determined and limited experimental studies have been conducted. It has been reported that viral diseases could be minimized by adjusting immune system through interaction with molecules such as peptidoglycans [43]. Additionally, postbiotics have been used as functional materials in various products such as dairy foods, vegetables, bread, meat, fish products, and food ingredients [38].

**Fig. 1:**
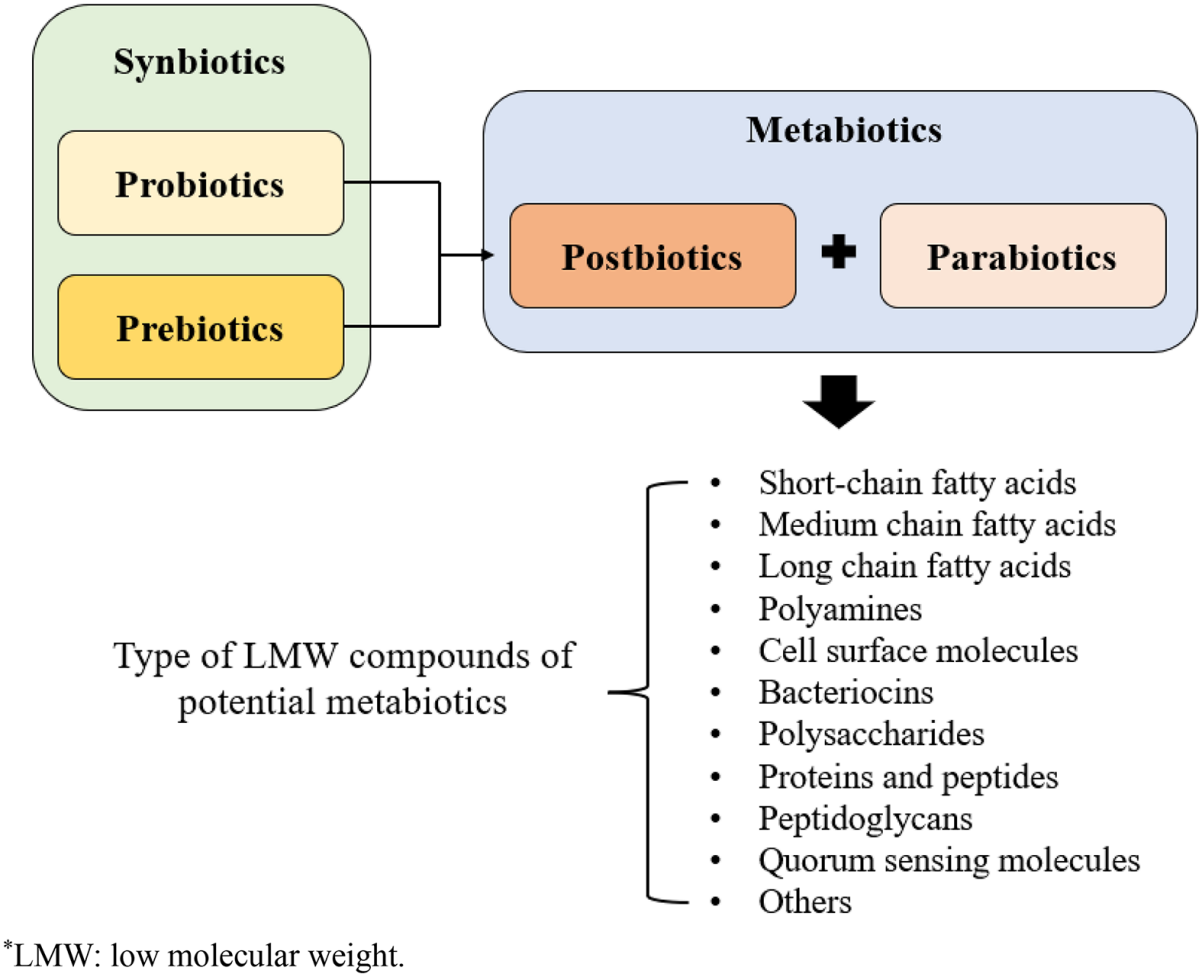
Composition and types of metabiotics.

Over the years, studies to identify microorganisms that benefit the host have been increasing. The functional efficacy of microorganisms is transmitted by living and non-living cells, and their metabolites. Thus, terms referred to in these studies have also been developed, including probiotics, synbiotics, postbiotics, and metabiotics [44]. Recently, a new concept for the probiotic term, metabiotics, has emerged. This has emerged as a new trend in biotechnology to improve the side effects of living probiotics and apply them in human treatments and health functional foods [45]. Metabiotics are characterized as bioactive substances produced through the metabolic activities of probiotic microorganism, contributing positively to a range of physiological process [7]. The term metabiotics is derived from the Greek prefix *meta*-including the meaning of change and transformation [7]. Over the past decade, scientific association have demonstrated that probiotics can form low-molecular weight (LMW) bioactive molecules by metabolizing complex food matrices and endogenous substances released by host cells [46]. Metabiotics contain active metabolites, signalling molecules and cells such as dead cells and their fragments after metabolizing [8]. Various type of metabiotics that can be produced by probiotic microorganism include SCFAs, medium- and long chain fatty acids, bacteriocins, polyamines, cell surface molecules, polysaccharides, peptidoglycans, protein and peptides, and quorum sensing molecules [47]. Fig. 1 represents an overview of metabiotics and their different types. Metabiotics are known to be more suitable and convenient for the long-term storage of functional products and are easier to administer or regulate than traditional probiotics. Metabiotics comprise a wide range of functional compounds with various beneficial effects on the host. They are also known to reduce oxidative stress, control blood pressure, immunomodulation, and exert anti-inflammatory and anticancer effects [45].

## Neurological Disorders Functionality by the Gut-Brain Axis

Recently, researchers have explored the health benefits associated with the composition of intestinal microorganisms. The gut microbiome plays a pivotal role in enhancing digestion and absorption of nutrients to generate energy [48]. Studies on the gut microbiome have highlighted the involvement of probiotics in various aspects of health, including the nervous system, inflammation, oncology, gastrointestinal tract, and endocrine system. Notably, probiotics plays a crucial role in supporting the immune system and brain health [49].

Furthermore, researchers have demonstrated a bidirectional association between the central nervous system (CNS) and gut microbiome [50]. Research indicates that microorganisms residing in the intestine release chemical messengers that affect cell responses along the vagus nerves, sending signals to the brain through these nerves [49]. Their main role is to regulate communication between the intestinal system and the CNS, now recognized as “gut-brain axis” [51].

Disruptions of the gut-brain axis can lead to neurological disorders such as depression, Alzheimer’s disease, and anxiety [51]. Intestinal bacteria are known to have the ability to produce numerous neuroactive molecules such as serotonin, gamma amino butyric acid (GABA) and SCFAs [52]. Recently, metabiotics, which is probiotic metabolites have recently been reported to possess neuroregulatory activity. Consequently, neurological disorders can be reduced through the delivery of substances produced by intestinal microbial communities, such as GABA, histamine, SCFAs, and serotonin [45, 51, 53, 54]. Table 1 shows metabiotics and associated microorganisms that control neurological disorders.

### Autism Spectrum Disorder

Autism spectrum disorder (ASD) is a brain development disorder characterized by difficulties in social interactions, language, and repetitive behavioral patterns [55]. The cause of ASD has not yet been indicated. However, the interaction between ASD and the gut-brain axis has received considerable attention [56]. As the association between gut microbiome and ASD has been reported, ASD research on the microbiota-gut-brain axis has been actively conducted [55]. In the autistic mouse model, *Bacteroides fragilis* and *Limosilactobacillus reuteri* improved communicative and behavioral disorders and reduced antisocial behavior [57]. In addition, SCFAs produced by various probiotic strains regulate ASD symptoms by increasing dopamine and catecholamine synthesis. Further research is needed to understand the interactions between microorganisms and autism [51].

### Anxiety and Depression

Increasing interest on the potential impact of the gut-brain axis on anxiety and depression, which are associated with mood or mental health disorders, has been observed. In particular, depression rates are expected to increase owing to coronavirus pandemic and post-pandemic conditions [58]. Recent studies suggests that the gut-brain axis, which involves bidirectional communication between the gut and the CNS, might play a pivotal role in the development and management of anxiety and depression [59]. Probiotics metabolites or metabiotics, are thought to influence this axis by modulating the production of neuroactive molecules, such as serotonin and GABA, both of which are involved in mental regulation [58]. In addition, the SCFAs produced by probiotics may have anti-inflammatory properties that can mitigate neuroinflammation, a factor implicated in mood and mental disorders. In addition, GABA, which is produced by *Lactobacillus* sp. and *Bifidobacterium*, helps reduce anxiety and depression by inhibiting acidic intestinal conditions [45]. Similarly, the administration of *L. rhamnosus* JB-1 led to a reduction in anxiety- and depression-related behaviors. These results were due to changes in GABA receptors in the brain and a decreased stress-induced corticosterone levels [51]. Additionally, the administration of *Lactobacillus plantarum* not only improved depressive behavior, but also enhanced learning abilities [60]. *Bacteroides fragilis* NCTC 9343 has been shown to reduce autistic behavioral responses by lowering the levels of 4-ethylphenylsulfate (4-EPS), a neurotoxic metabolite found in the serum that contributes to anxiety [47].

### Alzheimer’s and Parkinson’s Diseases

Alzheimer’s (AD) and Parkinson’s (PD) disease are neurodegenerative disorders that profoundly affect the nervous system and lead to progressive and debilitating symptoms. Primary neuropathological characteristics of AD are the accumulation of protein fragments, known as beta-amyloid (beta-amyloid plaques) outside neurons and aggregation of abnormally structured tau protein (tau tangles) inside neurons [61]. AD is primarily characterized by cognitive decline, memory impairment, and behavioral changes. In addition, AD has been reported as a major cause of dementia; however, the exact reason for remains unknown [62]. Recent studies have emphasized the role of the gut microbiome as a significant conditional factor influencing this disease. This has been demonstrated by extensive studies linking gut microbiota communities to neurodegenerative diseases, such as AD [63]. One study reported that SCFAs such as butyrate and propionate produced by *L. rhamnosus*, *L. reuteri*, and *B. fragilis*, regulate the cholinergic neuronal signaling and the anti-inflammatory pathways of the vagus nerves that connect to the brain, and this suppression mitigates AD [51]. In addition, *L. reuteri* ATG-F4 has been identified as a neuromodulatory metabolites, as it does not only increase the anti-inflammatory cytokine IL-10, but also increases serum dopamine levels [64]. In an AD mouse model with *B. breve* A1, an improvement in AD through the reduction of neuroinflammation induced by beta-amyloid peptides was observed, attributed to bacterial metabolite acetate [65].

PD is reported as one of the fastest growing neurological disorders globally due to various social and environmental factors and is the second most common neurodegenerative disorder [62, 66]. PD symptoms include motor, non-motor, emotional, and cognitive disorders, as well as inflammatory bowel syndrome [67]. This disease is idiopathic; however, the accumulation of alpha-synuclein and subsequent agitation of gastrointestinal function due to mucosal inflammation have been suggested as the main cause of PD development [51]. In addition, chronic gut inflammation results in systemic intestinal inflammation, neuroinflammation, and neurodegeneration, either through gut-vagus brain signaling or affecting the permeability of the blood-brain barrier [68]. Previous studies have shown that the ingestion of *L. plantarum* PS128 weakens motor dyskinesia, reduces dopamine neuronal apoptosis, increases striatal dopamine levels, and inhibits neuronal cell hyperactivation in 1-methyl-4-phenyl-1,2,3,6-tetrahydropyridine (MPTP)-induced PD mouse models [67]. *B. bifidum*, *B. longum*, *L. rhamnosus*, *L. rhamnosus* GG, *Lactococcus lactis* subsp. *lactis*, and *L. plantarum* LP28 effectively protect dopamine-releasing neurons and decrease motor dysfunction [69]. Moreover, ferulic acid, which has anti-inflammatory and antioxidant effects, has been confirmed to be effective in controlling PD by preventing lipid and protein oxidation and reducing the production of pro-inflammatory cytokines. SCFAs supplementation or intestinal microbiota reconstruction can alleviate PD symptoms [70] The above reports provide evidence that probiotic metabolites can potentially mitigate PD by addressing problems related to intestinal dysfunction, microbial spread, neurodegeneration prevention, and neuroinflammation alleviation [51].

## Immunomodulation and Anticancer Properties

Inflammation and cancer complement each other and are interrelated. Cancer is a serious diseases caused by chronic inflammation. Chronic and uncontrolled inflammation can promote cancer development owing to epigenetic changes, genomic instability, proliferation increase, and apoptosis resistance [71]. Metabiotics substances related to inflammation and cancer prevention and their functionality are shown in Table 2.

Studies reported that immunomodulatory mechanisms can alleviate, prevent, and treat autoimmune diseases and inflammation, and eliminate malignant host cells [45]. Metabiotics have been reported to exert several immunomodulatory effects. One research has reported that SCFAs maintain balance by reducing inflammatory cytokines such as IL-6 and TNF-α, stimulating the anti-inflammatory cytokine, IL-10 [71]. In addition, exopolysaccharides (EPS), monolayer proteins, peptidoglycans, lipoteichoic acid (LTA), conjugated linoleic acid, and peptides derived from probiotics have been reported to modulate the immune system [71]. Another research has reported polysaccharide-peptidoglycan complex by *Lactobacillus casei* inhibited IL-6 synthesis through the inhibition of nuclear factor kappa B (NF-κB) [72]. In addition, capsular polysaccharides (CPS) produced by *Bacillus fragilis* have been reported to modulate both innate and adaptive immunity [51]. LTA, a cell wall component of *Lactobacillus pararcasei* D3-5, showed anti-inflammatory effect by modulation the NF-κB signaling pathway. Additionally, metabiotics, including folate, acetate, propionate, and butyrate, are reported to regulate immunity by inhibiting histone deacetylases and acetylation [45]. Therefore, the efficacy of various metabiotics in regulating inflammation, mainly by upregulating the synthesis of anti-inflammatory cytokines and downregulating inflammatory mediators has been established, using cell lines, mouse models and even human experiments [71].

Gut microbiota or their metabolites are known to prevent various cancers. Among metabolic byproducts, SCFAs are widely acknowledged as potent anticancer components. Their primary focus was on histone deacetylases, which induce epigenetic alterations through posttranslational modifications of histone proteins [45]. Metabiotics *L. rhamnosus* MD14 containing SCFAs showed inhibition of colorectal carcinogenesis through Wnt/β-catenin signal pathway by reducing oncogenes such as NF-κB, COX-2, K-ras, and β-catenin and increasing tumor suppressor such as p53 gene [73]. Additionally, SCFAs produced by *Propionibacterium freudenrechii* have shown anticancer effects on gastric cell lines [51]. Similarly, butyrate derived from *L. plantarum* was estimated to have anti-colon cancer effect by confirming their capability inhibiting HT-29 cells growth [74]. The peptide produced by *Saccharomyces cerevisiae* CNCM I-3856 showed anti-colorectal cancer effects [75]. The anticancer effect of metabiotic cell-free supernatant (CFS) has also been reported. The CFS of *Lactobacillus acidophilus* 36YL has reported anti-canner effects on cervical cancer as well as anti-colon cancer cell lines [76]. Similarly, the CFS of *B. adolescentis* SPM0212 showed anti-colon cancer effects [51]. Furthermore, bacteriocins produced by *Enterococcus faecium*, *Bacillus subtilis*, and *Bifidobacterium* has been reported that gastric cancer is reduced by inhibiting the growth of *Helicobacter pylori* [51]. The ability of metabiotics to regulate cell differentiation, induce apoptosis, arrest cell cycle, and regulate epigenetic activity can be used to treat various malignant tumors [45]. Reports of various metabolites that have anticancer effects are available; however, more research to identify metabiotics that have anticancer effects, including new metabolites components or any other sources is required.

## Conclusion and Prospects

Probiotics have progressively developed in various form such as prebiotics, synbiotics, postbiotics, and metabiotics. General outlook on metabiotics, which are considered the next-generation probiotics, is highly promising and has significant potential in the realm of health and well-being.

As research on the connection between the gut and brain neurological health increases, research on the gut-brain axis is expected to be particularly interesting. Research has shown that metabiotics can play a pivotal role in brain function, neuroinflammation, and even the management of conditions such as ASD. In addition, new substances derived from probiotics and metabiotics are need for further material demonstration and human clinical studies of anticancer effects caused by chronic inflammation.

In the future, prevention and therapy using metabiotics is expected. Metabiotics related to intestinal microflora will not only improve the well-being of society but also demonstrate potential efficacy in the treatment and prevention of neurological disorders, as well as immunomodulatory and anticancer effects.

## Figures and Tables

**Table 1:** Metabiotics related with microorganisms and their neurological properties.

Components	Microorganism	Health functional properties	References
Dopamine	*Lactobacillus reuteri* ATG-F4	Increase of cytokine IL-10 and serum dopamine level (AD)	[66]
Dopamine	*Lactobacillus plantarum* PS128	Reduction of motor dyskinesia and neurol apoptosis (PD)	[69]
Dopamine	*Bifidobacterium bifidum*, *Bifidobacterium longum*, *Lactobacillus rhamnosus*, *Lactobacillus rhamnosus* GG, *Lactococcus lactis* subsp. *lactis*, *Lactobacillus plantarum* LP28	Protection of dopamine-releasing neurons Decrease of motor dysfunctions	[71]
GABA	*Lactobacillus* sp., *Bifidobacterium* sp.	Reduction of anxiety and depression (anxiety and depression)	[53]
GABA	*Lactobacillus rhamnosus* JB-1	Alleviation of anxiety and depression by decreasing corticosterone (anxiety and depression)	[8]
GABA	*Lactobacillus rhamnosus* YS9	Control nerve	[57]
Histamine	*Lactobacillus* sp., *Lactococcus* sp., *Enterococcus* sp., *Streptococcus* sp., *Escherichia* sp.	Regulation of emotions Regulation of sleep and cognitive function (cognition)	[53]
Metabolites	*Bacteroides fragilis* NCTC 9343	Reduction of autistic behavior (AD)	[49]
Metabolites	*Bifidobacterium breve* A1	Reduction of neuroinflammation (AD)	[67]
SCFA	*Lactobacillus rhamnosus*, *Lactobacillus reuteri*, *Bacterioides fragilis*	Regulation of anti-inflammatory pathways (AD)	[53]
Serotonin	*Lactobacillus* sp. *Lactococcus* sp., *Enterococcus* sp., *Streptococcus* sp., *Escherichia* sp.	Regulation of emotions, sleep, and cognitive function (anxiety and depression, cognition)	[53]

GABA, gamma-amino butyric acid; SCFAs, short-chain fatty acids; AD, Alzheimer's disease; PD, Parkinson’s disease.

**Table 2:** Metabiotics related with microorganism and their immunomodulation and anticancer properties.

Components	Microorganism	Health functional properties	References
Immunomodulation			
Capsular polysaccharides	*Bacillus fragilis*	Help innate and adaptive immunomodulation	[53]
Folate, acetate, propionate, and butyrate	*Lactobacillus plantarum*, *Lactobacillus casei*, *Bifidobacterium bifidum*, *Bifidobacterium infantis*, *Bifidobacterium longum*	Regulation of immunity through inhibition of histone deacetylases and histone acetylation	[49]
Lipoteichoic acid	*Lactobacillus paracasei* D3-5	Anti-inflammatory effect by regulation the nuclear factor-kB (NF-κB) signaling	[8]
Polysaccharide-peptidoglycan complex	*Lactobacillus casei*	Inhibition of IL-6 synthesis by inhibiting NF-κB	[74]
Anticancer			
Bacteriocin	*Enterococcus faecium*, *Bacillus subtilis*, *Bifidobacterium*	Inhibition of *Helicobacter pylori* growth (gastric cancer)	[53]
Butyrate	*Lactobacillus plantarum*	Anti-proliferative and apoptosis (colorectal cancer)	[76]
Cell-free supernatant	*Lactobacillus acidophilus* 36YL	Cytotoxic effects in HT-29 and HeLa cell lines (colorectal cancer and cervical cancer)	[78]
	*Bacillus adolescentis* SPM0212	Inhibition of cancer cell growth (colorectal cancer)	[53]
Lipoteichoic acid (LTA)	*Lactobacillus rhamnosus* GG	Preventing of skin cancer development	[49]
Peptide	*Saccharomyces cerevisiae* CNCM I 3856	Modification of pro-inflammatory cytokine production (colorectal cancer)	[77]
SCFAs	*Propionibacterium freudenreichii*,	Apoptosis or necrosis against gastric cancer	[53]
	*Lactobacillus rhamnosus* MD14	Preventing of colorectal cancer by targeting on the Wnt/β-Catenin pathway (colorectal cancer)	[9]

SCFAs, short-chain fatty acids.

## References

[1] Rasika DMD, Vidanarachchi JK, Luiz SF, Azeredo DRP, Cruz AG, Ranadheera CS (2021). Probiotic delivery through non-dairy plantbased food matrices. Agriculture.

[2] Kim KT, Yang SJ, Paik HD (2021). Probiotic properties of novel probiotic *Levilactobacillus brevis* KU15147 isolated from radish kimchi and its antioxidant and immune-enhancing activities. Food Sci. Biotechnol..

[3] Lu K, Dong S, Wu X, Jin R (2021). Probiotics in cancer. Front. Oncol..

[4] Sotoudegan F, Daniali M, Hassani S, Nikfar S, Abdollahi M (2019). Reappraisal of probiotics' safety in human. Food Chem. Toxicol..

[5] Lee NK, Paik HD (2021). Prophylactic effects of probiotics on respiratory viruses including COVID-19: a review. Food Sci. Biotechnol..

[6] Hong SW (2020). Postbiotics: next-generation of lactic acid bacteria. Livest. Food Sci. Ind..

[7] Oleskin AV, Shenderov BA (2019). Probiotics and psychobiotics: the role of microbial neurochemicals. Probiotics Antimicrob. Proteins.

[8] Pihurov M, Pacularu-Burada B, Cotarlet M, Vasile MA, Bahrim GE (2021). Novel insight for metabiotics production by using artisanal probiotic cultures. Microorganisms.

[9] Sharma M, Shukla G (2016). Metabiotics: one step ahead of probiotics; an insight into mechanisms involved in anticancerous effect in colorectal cancer. Front. Microbiol..

[10] Kapoor B, Singh A, Gulati M, Singh SK, Rani P, Alzahrani Q (2022). Orchestration of obesolytic activity of microbiome: metabiotics at centre stage. Curr. Drug Metab..

[11] Zommiti M, Feuilloley MGJ, Connil N (2020). Update of probiotics in human world: a nonstop source of benefactions till the end of time. Microorganisms.

[12] Son SH, Yang SJ, Jeon HL, Yu HS, Lee NK, Park YS (2018). Antioxidant and immunostimulatory effect or potential probiotic *Lactobacillus paraplantarum* SC61 isolated from korean traditional fermented food, *jangajii*. Microb. Pathog..

[13] Lee NK, Kim WS, Paik HD (2019). *Bacillus* strains as human probiotics: characterization, safety, microbiome, and probiotic carrier. Food Sci. Biotechnol..

[14] Bottari B, Castellone V, Neviani E (2021). Probiotics and COVID-19. Int. J. Food Sci. Nutr..

[15] Jeon HL, Yang SJ, Son SH, Kim WS, Lee NK, Paik HD (2018). Evaluation of probiotic *Bacillus subtilis* P229 isolated from *cheonggukjang* and its application in soybean fermentation. LWT-Food Sci. Technol..

[16] Slizewska K, Markowiak-Kopec P, Slizewska W (2021). The role of probiotics in cancer prevention. Cancers.

[17] Stavropoulou E, Bezirtzoglou E (2020). Probiotics in medicine: a long debate. Front. Immunol..

[18] Singh K, Rao A (2021). Probiotics: a potential immunomodulation in COVID-19 infection management. Nutr. Res..

[19] Pique N, Berlanga M, Minana-Galbis D (2019). Health benefits of heat-killed (Tyndallized) probiotics: an overview. Int. J. Mol. Sci..

[20] Zuo F, Marcotte H (2021). Advantage mechanistic understanding and bioengineering of probiotic *lactobacilli* and *bifidobacteria* by genome editing. Curr. Opin. Biotechnol..

[21] Guan Q, Xiong T, Xie M (2021). Influence of probiotic fermented fruit and vegetables on human health and the related industrial development trend. Engineering.

[22] Kechagia M, Basoulis D, Konstantopoulou S, Dimitriadi D, Gyftopoulou K, Skarmoutsou N (2013). Health benefits of probiotics: a review. ISRN Nutr..

[23] De Simone C (2019). The unregulated probiotic market. Clin. Gastroenterol. Hepatol..

[24] Gibson GR, Roberfroid MB (1995). Dietary modulation of the human colonic microbiota: introducing the concept of prebiotics. J. Nutr..

[25] Yadav MK, Kumari I, Singh B, Sharma KK, Tiwari SK (2022). Probiotics, prebiotics and synbiotics: safe options for next-generation therapeutics. Appl. Microbiol. Biotechnol..

[26] Gibson GR, Hutkins R, Sanders ME, Prescott SL, Reimer RA, Salminen SJ (2017). Expert consensus document: the international scientific association for probiotics and prebiotics (ISAPP) consensus statement on the definition and scope of prebiotics. Nat. Rev. Gastroenterol. Hepatol..

[27] Oniszczuk A, Oniszczuk T, Gancarz M, Szymanska J (2021). Role of gut microbiota, probiotics and prebiotics in the cardiovascular diseases. Molecules.

[28] Farias DP, Araujo FF, Neri-Numa IA, Pastore GM (2019). Prebiotics: trends in food, health and technological applications. Trends Food Sci. Technol..

[29] Mohanty D, Misra S, Mohapatra S, Sahu PS (2018). Prebiotics and synbiotics: recent concepts in nutrition. Food Biosci..

[30] Davila I, Gullon B, Alonso JL, Labidi J, Gullon P (2019). Vine shoots as new source for the manufacture of prebiotic oligosaccharides. Carbohydr. Polym..

[31] Plamada D, Vodnar DC (2021). Polyphenols-Gut microbiota interrelationship: a transition to a new generation of prebiotics. Nutrients.

[32] Sanders ME, Merenstein DJ, Reid G, Gibson GR, Rastall RA (2019). Probiotics and prebiotics in intestinal health and disease: from biology to the clinic. Nat. Rev. Gastroenterol. Hepatol..

[33] Martyniak A, Medynska-Przeczek A, Wedrychowicz A, Skoczen S, Tomasik PJ (2021). Prebiotics, probiotics, synbiotics, parabiotics and postbiotics compounds in IBD. Biomolecules.

[34] Nunez-Sanchez M, Herisson FM, Cluzel GL, Caplice NM (2021). Metabiolic syndrome and symbiotic targeting of the gut microbiome. Curr. Opin. Food Sci..

[35] Swanson KS, Gibson GR, Hutkins R, Reimer RA, Reid G, Verbeke K (2020). The international scientific association for probiotics and prebiotics (ISAPP) consensus statement on the definition and scope of synbiotics. Nat. Rev. Gastroenterol. Hepatol..

[36] Mofid V, Izadi A, Mojtahedi SY, Khedmat L (2020). Therapeutic and nutritional effects of symbiotic yogurts in children and adults: a clinical review. Probiotics Antimicrob. Proteins.

[37] Seong G, Lee S, Min YW, Jang YS, Park SY, Kim CH (2020). Effect of symbiotic containing *Lactobacillus paracasei* and *Opuntia humifusa* on a murine model of irritable bowel syndrome. Nutrients.

[38] Sharma N, Kang DK, Paik HD, Park YS (2023). Beyond probiotics: a narrative review on an era of revolution. Food Sci. Biotechnol..

[39] Salminen S, Collado MC, Endo A, Hill C, Lebeer S, Quigley EMM (2021). The international scientific association of probiotics and prebiotics (ISAPP) consensus statement on the definition and scope of postbiotics. Nat. Rev. Gastroenterol. Hepatol..

[40] Wegh CAM, Geerlings S, Knol J, Roeselers G, Belzer C (2019). Postbiotics and their potential applications in early life nutrition and beyond. Int. J. Mol. Sci..

[41] Teame T, Wang A, Xie M, Zhang Z, Yang Y, Ding Q (2020). Paraprobiotics and postbiotics of probiotic Lactobacilli their positive effects on the host and action mechanisms: a review. Front. Nutr..

[42] Rather IA, Choi SB, Kamli MR, Hakeem KR, Sabir JSM, Park YH (2021). Potential adjuvant therapeutic effect of *Lactobacillus plantarum* Probio-88 postbiotics against SARS-COV-2. Vaccines.

[43] Xavier-Santos D, Padilha M, Fabiano GA, Vinderola G, Cruz AG, Sivieri K (2022). Evidences and perspectives of the use of probiotics, prebiotics, synbiotics, and postbiotics as adjuvants for prevention and treatment of COVID-19: a bibliometric analysis and systematic review. Trends Food Sci. Technol..

[44] Nataraj BH, Shivanna SK, Rao P, Nagpal R, Behare PV (2021). Evolotionary concepts in the functional biotics arena: a mini-review. Food Sci. Biotechnol..

[45] Biswas I, Das Mohapatra PK (2023). Recent advancement in metabiotics: a consortium with bioactive molecules after fermentation by probiotic bacteria with multidisciplinary application potential and future solution in health sector. Bioresour. Technol..

[46] Shenderov BA (2013). Metabiotics: novel idea or natural development of probiotic conception. Microb. Ecol. Health Dis..

[47] Singhal B, Chaudhary N, Verma DK, Patel AR, Sandhu KS, Baldi A, Garcia S (2021). Metabiotics as functional metabolites of probiotics: an emerging concept and its potential application in food and health. Biotechnical processing in the food industry: New methods, techniques, and applications, 1st Ed.

[48] Dalton A, Mermier C, Zuhl M (2019). Exercise influence on the microbiome-gut-brain axis. Gut Microbes.

[49] Dahiya D, Nigam PS (2022). Probiotics, prebiotics, synbiotics, and fermented food as potential biotics in nutrition improving health via microbiome-gut brain axis. Fermentation.

[50] Bauer KC, Rees T, Finlay BB (2019). The gut microbiota-brain axis expands neurologic function: a nervous rapport. BioEssays.

[51] Singh A, Vishwakarma V, Singhal B (2018). Metabiotics: the functional metabolic signatures of probiotics: current state-of-art and future research priorities. Adv. Biosci. Biotechnol..

[52] Caputi V, Giron MC (2018). Microbiome-gut brain axis and toll-like receptors in Parkinson's disease. Int. J. Mol. Sci..

[53] Snigdha S, Ha K, Tsai P, Dinan TG, Bartos JD, Shahid M (2022). Probiotics: potential novel therapeutics for microbiota-gut-brain axis dysfunction across gender and lifespan. Pharmacol. Ther..

[54] Suda K, Matsuda K (2022). How microbes affect depression: underlying mechanisms via the gut-brain axis and the modulating role of probiotics. Int. J. Mol. Sci..

[55] Li Q, Zhou JM (2016). The microbiota-gut-brain axis and its potential therapeutic role in autism spectrum disorder. Neuroscience.

[56] Mayer EA, Tillisch K, Gupta A (2015). Gut/brain axis and the microbiota. J. Clin. Invest..

[57] Varela-Trinidad GU, Dominguez-Diaz C, Solorzano-Castanedo K, Iniguez-Gutierrez L, Hernandez-Flores TJ, Fafutis-Morris M (2022). Probiotics: protecting our health from the gut. Microorganisms.

[58] Jach ME, Serefko A, Szopa A, Sajnaga E, Golczyk H, Santos LS (2023). The role of probiotics and their metabiotics in the treatment of depression. Molecules.

[59] Chen Y, Xu J, Chen Y (2021). Regulation of neurotransmitters by the gut microbiota and effects on cognition in neurological disorders. Nutrients.

[60] Zou R, Tian P, Xu M, Zhe H, Zhao J, Zhang H (2021). Psychobiotics as a novel strategy for alleviating anxiety and depression. J. Funct. Food..

[61] Szandruk-Bender M, Wiatrak B, Szelag A (2022). The risk of developing Alzheimer's disease and Parkinson's disease in patients with inflammatory bowel disease: a meta-analysis. J. Clin. Med..

[62] Oroojzadeh P, Bostanabad SY, Lorfi H (2022). Psychobiotics: the influence of gut microbiota the gut-brain axis in neurological disorders. J. Mol. Neurosci..

[63] Chen J, Chen DF, Cho KS (2023). The role of gut microbiota in glaucoma progression and other retinal diseases. Am. J. Pathol..

[64] Beck BR, Park GS, Jeong DY, Lee YH, Im S, Song WH (2019). Multidisciplinary and comparative investigations of potential psychobiotic effects of *Lactobacillus* strains isolated from newborns and their impact on gut microbiota and ileal transcriptome in a healthy murine model. Front. Cell. Infect. Microbiol..

[65] Dasriya VLD, Samtiya M, Dhewa T, Puniya M, Kumar S, Ranveer S (2022). Etiology and management of Alzheimer's disease: potential role of gut microbiota modulation with probiotics supplementation. J. Food Biochem..

[66] Tans AH, Lim SY, Lang AE (2022). The microbiome-gut-brain axis in Parkinson disease-from basic research to the clinic. Nat. Rev. Neurol..

[67] Chu C, Yu L, Li Y, Guo H, Zhai Q, Chen W (2023). Meta-analysis of randomized controlled trials of the effects of probiotics in Parkinson's disease. Food Funct..

[68] Metta V, Leta V, Mrudula KR, Prashanth LK, Goyal V, Borgohain R (2022). Gastrointestinal dysfunction in Parkinson's disease: molecular pathology and implication of gut microbiome, probiotics, and fecal microbiota transplantation. J. Neurol..

[69] Hsieh TH, Kuo CW, Hsieh KH (2020). Probiotics alleviate the progressive deterioration of motor functions in a mouse model of Parkinson's disease. Brain Sci..

[70] Zheng SY, Li HZ, Xu RC, Miao WT, Dai MY, Ding ST (2021). Potential roles of gut-microbiota and microbial metabolites in Parkinson's disease. Ageing Res. Rev..

[71] Sharma M, Shukla G (2016). Metabiotics: one step ahead of probiotics; an insight into mechanism involved in anticancerous effect in colorectal cancer. Front. Microbiol..

[72] Matsumoto S, Hara T, Nagaoka M, Mike A, Mitsuyama K, Sako T (2008). A component of polysaccharide peptidoglycan complex on *Lactobacillus* induced an improvement of murine model of inflammatory bowel diseases and colitis-associated cancer. Immunology.

[73] Sharma M, Shukla G (2020). Administration of metabiotics extracted from probiotic *Lactobacillus rhamnosus* MD14 inhibit experimental colorectal carcinogenesis by targeting Wnt/β-Catenin pathway. Front. Oncol..

[74] Kim HJ, An J, Ha EM (2022). *Lactobacillus plantarum*-derived metabolites sensitize the tumor-suppressive effects of butyrate by regulating the functional expression of SMCT1 in 5-FU-resistant colorectal cancer cells. J. Microbiol..

[75] Zanello G, Berri M, Dupont J, Sizaret PY, Dlnca R, Salmon H (2011). *Saccharomyces cerevisiae* modulates immune gene expression and inhibits ETEC-mediated ERK1/2 and p38 signaling pathways in intestinal epithelial cells. PLoS One.

[76] Nami Y, Abdullah N, Haghshenas B, Radiah D, Rosli R, Khosroushahi AY (2014). Probiotic potential and biotherapeutic effects of newly isolated vaginal *Lactobacillus acidophilus* 36YL strain on cancer cells. Anaerobe.

